# Optimisation of Strength Properties of FDM Printed Parts—A Critical Review

**DOI:** 10.3390/polym13101587

**Published:** 2021-05-14

**Authors:** Daniyar Syrlybayev, Beibit Zharylkassyn, Aidana Seisekulova, Mustakhim Akhmetov, Asma Perveen, Didier Talamona

**Affiliations:** Department of Mechanical & Aerospace Engineering, Nazarbayev University, Nur-Sultan 010000, Kazakhstan; daniyar.syrlybayev@nu.edu.kz (D.S.); beibit.zharylkassyn@nu.edu.kz (B.Z.); aidana.seisekulova@nu.edu.kz (A.S.); mustakhim.akhmetov@nu.edu.kz (M.A.)

**Keywords:** strength, laser preprocessing, polymer materials

## Abstract

Additive Manufacturing is currently growing fast, especially fused deposition modeling (FDM), also known as fused filament fabrication (FFF). When manufacturing parts use FDM, there are two key parameters—strength of the part and dimensional accuracy—that need to be considered. Although FDM is a popular technology for fabricating prototypes with complex geometry and other part product with reduced cycle time, it is also limited by several drawbacks including inadequate mechanical properties and reduced dimensional accuracy. It is evident that part qualities are greatly influenced by the various process parameters, therefore an extensive review of the effects of the following process parameters was carried out: infill density, infill patterns, extrusion temperature, layer thickness, nozzle diameter, raster angle and build orientation on the mechanical properties. It was found from the literature that layer thickness is the most important factor among the studied ones. Although manipulation of process parameters makes significant differences in the quality and mechanical properties of the printed part, the ideal combination of parameters is challenging to achieve. Hence, this study also includes the influence of pre-processing of the printed part to improve the part strength and new research trends such as, vacuum-assisted FDM that has shown to improve the quality of the printing due to improved bonding between the layers. Advances in materials and technologies that are currently under development are presented. For example, the pre-deposition heating method, using an IR lamp of other technologies, shows a positive impact on the mechanical properties of the printed parts.

## 1. Introduction

The basic principle of product manufacturing includes the subtractive, formative, and additive manufacturing (AM) process. Among these three categories, additive manufacturing is a relatively newer technology which uses a deposition of material layer by layer for the fabrication of parts using a computer-aided designed model. The basic principle involved in the additive manufacturing process is the generation of a virtual model in a computer, then slicing this model into 2D cross-sections and translating these 2D data to the AM machine in order to manufacture the physical product layer by layer [1,2]. Li et al. (2018) classified AM under several categories: polymerization, material jetting, binder jetting, materials extrusion, sheet lamination, powder bed fusion and directed energy deposition [3]. Among all these AM techniques, the fused deposition modeling under the category of materials extrusion has received the most consumer interest, attention, development, and innovation throughout the last few decades. In this process, a nozzle containing molted filament can move in a 2D plan to create one layer of a cross-section of a whole part, and the built platform is able to move up or down for each layer [4].

Although FDM was originally patented by Crump in 1988 who started the company Stratasys in 1989, this patent has now expired and therefore there are possibilities for low-cost FDM printers and this manufacturing process is gaining more and more attention from consumers. In recent years, numerous applications of FDM technology have been developed due to their simplicity and low cost. Therefore, there are expectations of high part quality and high performance for this technology. Numerous researchers worldwide have contributed to the development of the FDM process and its high performance. Turner et al. (2014) [5] presented a review study on process design as well as modeling of FDM and subsequently, Turner et al. (2015) [6] studied the roughness and dimensional accuracy of printed parts. Popescu, et al. (2018) [7] investigated various feedstock materials along with their mechanical materials properties. Vyavahare (2019) [8] presented an overview of the FDM process. Cuan-Urquizo et al. (2019) [9] studied computation and analytical methods to predict the mechanical properties. Relevant data detailing the strength improvement of FDM parts are scarce in the literature. Therefore, this study intends to conduct a concise review of work in terms of improving the strength properties of FDM products. This study starts with an overview of the FDM process and its applications. Then, effects of the process parameters on the strength are discussed in Section 2. Section 3 looks at the pre-processing techniques available to improve the strength of parts. Section 4 presents new developments in materials and technology such as the vacuum-assisted FDM technique.

### 1.1. Overview of FDM Process

**Figure 1:**
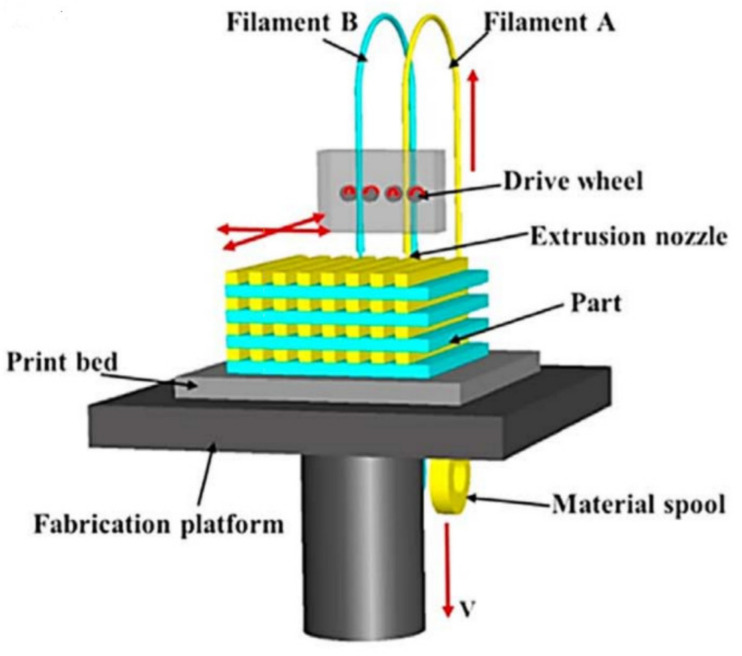
Schematic diagrams of fused deposition modelling [15].

FDM uses thermoplastic materials to print parts layer by layer. Usually, a (or several) continuous filament made of thermoplastic polymer is heated to the point of viscous state and extruded layer by layer on a platform to build the part (Figure 1). The thermo-plasticity properties of the material assist the fusing of layers together as well as the solidification process while the temperature is decreasing. Bonding between the layers will be generated due to the interaction of the molecules when one layer stays in a molten state and bound to the solid layer while solidifying. However, voids might appear between the layers due to the fast freezing of molten fibers or the lack of overlap between the extruded material and the solidified material. Therefore, these bonds exhibit mechanical properties unlike those created by the conventional manufacturing process and therefore it is difficult to predict the mechanical properties of FDM printed parts [10]. It was reported that layer thickness, layer width and infill orientation/density play a critical role in the mechanical properties of the part produced. Although FDM offers on the one hand cost reduction, faster production, and the simplest work setup, on the other hand, bad surface quality, weaker mechanical properties, visibility of layer thickness are some of the limitations associated with FDM [11,12]. In the literature, it is suggested that interlayer distortion is the reason behind this weaker mechanical property [13]. Some studies have reported improved mechanical properties by using fiber-reinforced composites, however, challenges involving fiber orientation, void formation, improper bonding between fiber and matrix are still to be investigated and addressed [14,15].

### 1.2. Bond Formation Mechanism

As the FDM part contains partially bonded cylindrical filaments of build materials, this bonding quality, the extent of inter-layer bonding and intralayer bonding play an imperative part in deciding the mechanical properties of the produced part. During solidification, filaments create some sort of bridging via viscous sintering between two subsequent filaments which is known as the neck. As per literature, part strength is regulated by the intra-layer bond, inter-layer bond as well as neck size. Frenkel (1945) [16] proposed a model which takes into consideration the surface tension and viscous flow to explain viscous sintering of polymer spherical crystalline particles. Based on this model, Bellehumeur, et al. (2004) [17] conducted a thermal study of the FDM process providing an estimation of cooling profiles and bond formation. Sun et al. (2008) [18] concluded that surface tension plays a critical role in the partial coalescence of materials due to intra-layer and inter-layer bonding. Rodriguez, et al. (2001) [19] suggested that the presence of void and lack of molecular orientation results in decreased strength as well as decreased elastic modulus. Anna and Guceri (2003) [20] concluded the importance of build direction in determining the extent of neck formation which then influences shrinkage and strength. Pavan, et al. (2014) [21] modified the model of Frenkel (1945) and Pokluda, et al. (1997) [22] for cylindrical filament which ascertains time dependence nature of neck size between two subsequent filaments where effective neck area is derived using a number of neck formation that occurs within the part. Then the strength of the printed part can be estimated by using load carried by this effective neck area. Several mechanisms have been reported to be responsible for this neck development which include viscous sintering and diffusion. Minimization of the free energy seems to be the motive of sintering the adjacent layer which then results into reduced surface area and this gained energy due to surface reduction dissipates via viscous flow. As reported by Frenkel (1945), viscous sintering plays crucial part in neck growth mechanism. In addition, higher processing temperature compared to glass transition temperature is a prerequisite for fusion bonding occurrence between thermoplastic polymers. This bond formation comprises of two subsequent steps such as surface contact and intermolecular diffusion of polymer segments across the wetted surface. Consequently, formation of this wetted interface as well as the degree of inter-molecular diffusion decide the strength of the formed fusion bond between two polymers [23].

### 1.3. Application of FDM Products

FDM technology is one of the most popular AM technologies that permits the fabrication of components that cannot be manufacture with conventional machining, are durable, and also made of high strength thermoplastic materials such as ULTEM, acrylonitrile butadiene styrene (ABS), polycarbonate, polyphenylsulfone, polylactic acid (PLA) [24,25,26,27]. Versatile applications requiring quick and inexpensive parts, prototypes, or rough and rigid parts for end users can be fabricated by FDM technology. FDM has also found applications in the Aerospace industry where traditional metal components are replaced by FDM fabricated parts to reduce weight and yet sufficiently robust. As a result of the deployment of FDM technology, the turnaround time for part repairment has been reduced as well. It was reported that Mars Rover from NASA had about 70 production-grade thermoplastic parts due to their light weight and durability properties to endure the difficult environment of space. FDM parts are also used in prototype applications requiring less surface finish as well as fewer resolutions since it offers cost reduction and requires no chemical postprocessing [28]. Recently, a low weight but high strength unmanned aerial vehicle (UAV) was reported to be fabricated by Stratasys and Aurora Flight Sciences using ULTEM 9085 material and FDM technology where honeycomb internal structure was used inside the internal wing design. Boeing uses FDM parts for its 777-300ER door handle and camera case. In addition, Moog Aircraft Group also adopted FDM to manufacture maintenance fixtures [25,29]. It was also reported that automobile intake manifolds for the Society of Automotive Engineers (SAE) were fabricated using FDM [30].

FDM also has also found applications in the medical industries [31] for personalized medicine. Evila, et al. [32] reported the fabrication of customized tracheal stents by using FDM technology which offers reduced cost and excellent surface. Reconstruction of the geometry and functionality of lumbar intervertebral disc implants also uses FDM technology [33]. In addition, FDM has been extensively used for scaffoldings and tissue engineering [34,35,36,37,38,39]. A list of typical FDM applications, but not exhaustive, is shown in Table 1.

## 2. Influence of Process Parameters on the Strength of FDM Parts

**Figure 2:**
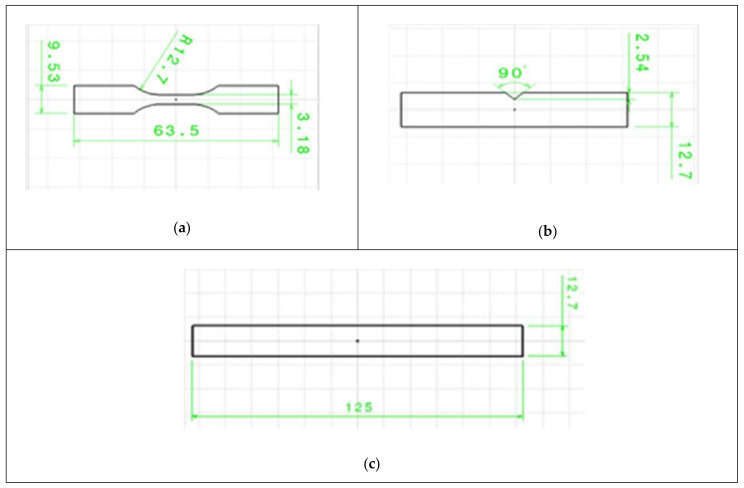
Standard specimens used for: (**a**) tensile test; (**b**) impact test; (**c**) flexural test [60].

In the USA, the testing of the mechanical properties of AM parts is regulated by ASTM standards. Special specimens are manufactured depending on the loading applied on the part. Figure 2 shows standard specimens used for tensile, flexural and impact strength testing of parts. Once the specimens have been manufactured, the testing is conducted according to the standard procedure until the part ruptures and the load–strain relationship is determined for each part. This relationship allows the establishment and further investigation of the part mechanical properties that are required for a specific application.

In general, the mechanical properties of the FDM printed parts can be improved by using a filament with high strength and by optimizing the process parameters (such as extrusion temperature, printing speed, infill density for example). The two most commonly used materials for the FDM are Polylactic Acid (PLA) and Acrylonitrile Butadiene Styrene (ABS). When comparing the ultimate tensile strength of these two materials, it is PLA that shows the highest UTS [61]. However, it was found that under certain combinations of the process parameters, parts manufactured from PLA can have low mechanical properties and can be even weaker than that parts made from ABS. Therefore, a substantial amount of research investigated the optimization of the process parameters of the FDM with the aim to maximize the mechanical properties.

### 2.1. Effect of Infill Density

Additive manufacturing allows manufacturing parts that are either solid or with a partial infill known as “infill density” that can vary from 0% (hollow part) to 100% (solid part). The infill density can have different patterns such as honeycomb or rectilinear for example. The load-bearing capacity of a part should increase when increasing the amount of the material inside the part. This finding was reported by Jatti et al. (2019) [60] during testing of flexural and tensile strengths of parts. A similar trend was observed by Ramkumar (2019) [62] who tested the impact resistance of the specimen using a standard IZOD test. The infill pattern was found to be a rank 1 parameter affecting the strength of the part [61,63,64] (Radhwan et al. (2019), Vicente et al. (2019), Rodriguez-Panes et al. (2018)) when a tensile test is done. Melenka et al. (2015) [65] found that the infill density is the most significant parameter determining the tensile strength and elastic modulus of the printed part.

**Figure 3:**
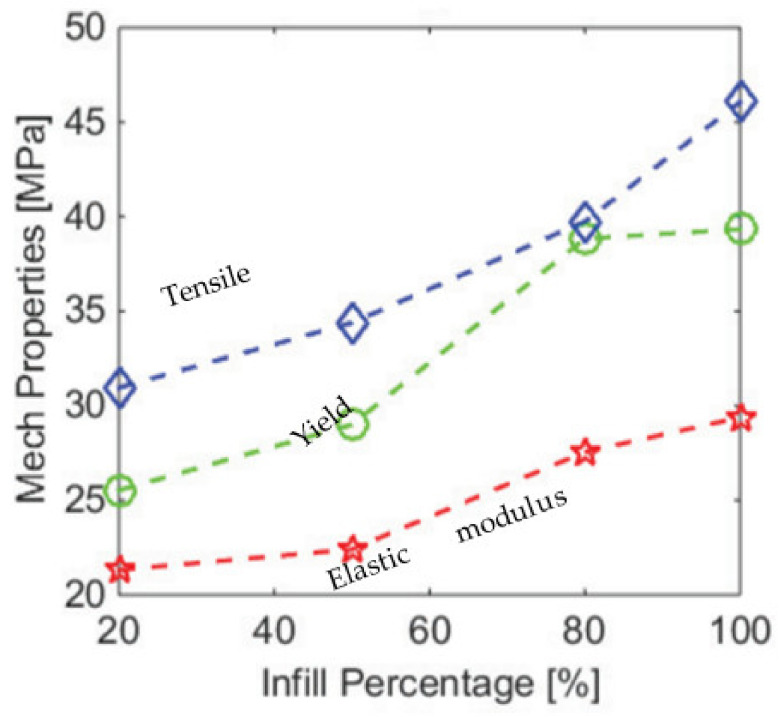
Dependence of the mechanical properties on the infill percent [66] (Alafaghani et al.).

Similar results were observed on Young’s modulus. Alafaghani et al. (2017) [66] measured the elastic modulus under the tensile loading of the PLA part using 0.02% offset method and found that when the infill percent is increased from 20% to 100%, the tensile stiffness increases gradually from 2 to 2.5 GPa as shown in Figure 3. The same trend was obtained by Vicente et al. (2019) [64], who conducted a tensile test on the ABS parts. When the infill percent was increased from 95 to 105% (negative airgap), the part strength was increased from 700 to 720 MPa. In general, it was found that the PLA filaments were stiffer than ABS.

**Figure 4:**
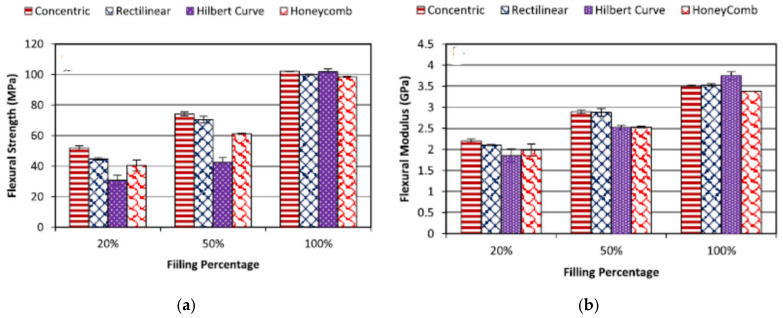
(**a**,**b**) Mechanical properties of the parts (Akhoundi, et al. 2019) [67].

The effect of the infill density may seem to be obvious, however, the abovementioned studies used the whole area of the cross-section to calculate the UTS. In fact, only part of the cross-section (which was infilled) bears the load as the cross-section was not solid (for infill smaller than 100%). Therefore, proper metrics should be developed to account for partial infill. To address this issue, Akhoundi et al. (2019) [67], investigated specific mechanical properties of parts tested and evaluated the mechanical properties of each part that were divided by their mass to account for the infill. Figure 4a,b shows the results for the flexural strengths as well as the corresponding stiffness modulus. It can be seen that the specific flexural mechanical properties (same properties divided by the mass of the part) are similar, which shows that the mechanical properties of the material are marginally affected by the infill density but the reduction of infill density will generate a decrease in the load-bearing capacity of the part. However, considering the tensile testing only, in most cases, the 50% infill is not as efficient as 100% and 20%. The specific tensile and flexural moduli are not affected to the extreme by the variation of the infill as the solid shell will provide the main strength of the part.

### 2.2. Effect of Infill Patterns

Apart from the infill density, the infill pattern also affects the mechanical properties of the part. The infill pattern determines how the infilled filaments interact with each other while subjected to loading.

**Figure 5:**
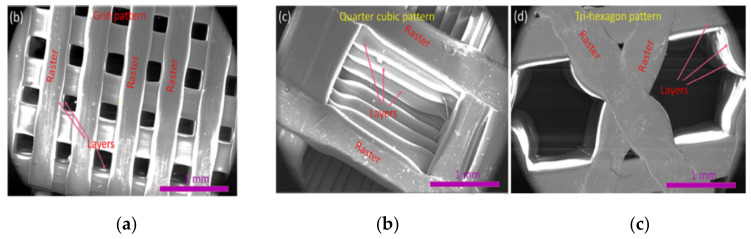
SEM images of various infill patterns: (**a**) Grid pattern; (**b**) Quarter cubic pattern; (**c**) Tri-hexagon pattern [68] (Alayaldi, et al. (2019)).

Alayoldi, et al. (2020) [68] investigated the effect of the infill pattern on the compressive strength of the part. It was found that triangular, grid and hexagonal infilled parts resulted in similar ultimate tensile strength (56–72 MPa), while the quarter cubic infill exhibited a significantly lower strength of 27 MPa. It was also found that the grid pattern had the highest tensile strength due to its special layer arrangement in which the infill layers are crisscrossed one above the other as shown in Figure 5. This was not observed for the case of quarter cubic pattern, in which there is an offset between the layers. When the part was loaded, this offset area was unsupported by the preceding layers and acted as a cantilever in bending. As a result, overall strength drops to 27 MPa.

It was also found that using patterns in which layers are located on one another tends to make the part more brittle and lower its impact strength. A study involving the impact characteristics of the FDM-ed parts showed that a rectilinear pattern in which all layers are aligned has poorer impact resistance compared to the honeycomb infill pattern. This happened because the crack propagation in the honeycomb pattern is problematic due to variant raster orientation at 0, 60 and 120 degrees.

Similar results were reported by Chadha, et al. (2019) [69] who studied how grid, triangular and honeycomb infill patterns perform under the flexural and tensile loadings. It was found that the triangular pattern has the highest strength followed by the grid and finally by honeycomb patterns under both bending and tension. SEM images of the fracture surfaces indicated that in the case of the grid pattern, printed filaments did not change its circular cross-section. This means that those filaments did not experience any necking, which might be the evidence of the brittle fracture. However, honeycomb and triangular patterns failed in a ductile manner and their filaments’ cross-section became oval due to the necking.

Akhoundi, et al. (2019) [67] suggested that in order to increase the tensile strength on the part, either all filaments should be aligned with the load application direction or the fusion between adjutant fibers should be increased. Fusion continues as long as the filament temperature cools down to glass transition temperature (Wool, et al. (1981)) [70]. Hilbert’s curve infill pattern is based on this principle. It can be seen from the results obtained by Akhoundi et al. [67] that the highest increase of the tensile specific strength was observed in the case of Hilbert’s curve infill pattern, which involves short paths. When one of these paths was deposited, it did not have much time to cool down before the other path was deposited next to it. Thus, increasing the infill to 100% yields a dramatic rise in the strength from 28 MPa at 20% infill to 60 MPa at 100%. In this case, the fusion of the adjutant paths has the dominant effect on the overall strength. On the other hand, concentric infill pattern, the one in which load application and filament deposition directions are aligned showed superior results at all infill densities.

The findings by Akhoundi, et al. (2019) regarding the honeycomb pattern contradict the findings of Cwikla, et al. (2017) [71] who investigated that the ABS part filled with honeycomb pattern shows commensurable strength with concentric infill and 40% density. The concentric pattern was not recommended for torsional applications, because due to its symmetrical geometry the torsional stiffness will fall. At the same time, traditional grid and rectilinear patterns have detrimental effects on mechanical properties.

Due to conflicting findings, it appears that further research is needed to evaluate how infill patterns influence the mechanical properties of AM parts. Additionally, as new infill patterns are available, there is a need to investigate their behavior under several loading conditions.

### 2.3. Effect of Extrusion Temperature

The extrusion temperature, if properly set, can have a positive effect on the mechanical properties of a part. For improved mechanical properties, fusion between the new layer and the existing layers takes place before the extruded filament cools down below its glass transition temperature, and the longer it stays at a higher temperature than its glass transition level, the better the bond becomes. This might explain the reason why the mechanical properties of PLA parts are superior to those of the ABS. PLA with lower glass transition temperature as well as lower heat conduction coefficient fuses better with the adjacent layers and filaments.

**Figure 6:**
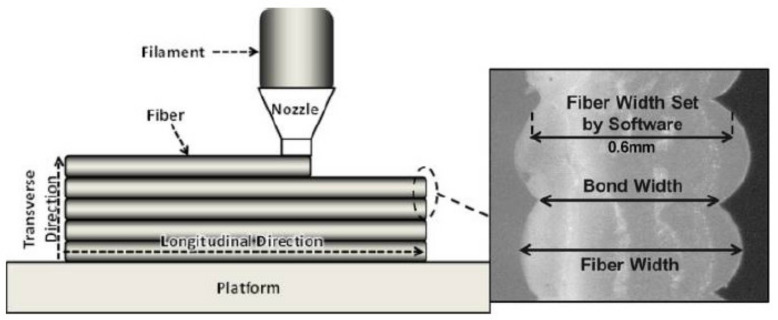
Bond width compared to fiber width [72] (Coogan, et al. (2016)).

The abovementioned correlation was verified by Coogan, et al. (2016) [72] who studied the bonding between extruded filaments. In their work, they printed hollow boxes with a thickness of one raster. Afterward, it was cut by a laser cutter to make test specimens. It was found that the increase in the extrusion temperature yields much stronger bonds. This happened due to increased wetting. At high temperatures, the fluidity of molten PLA increases, which facilitated the adhesion of the newly deposited PLA fiber on the extruded one and thus bond width between extruded raster increased which resulted in an increase in the strength as shown in Figure 6. Their study also concluded that in order to increase the bonding between filaments, wetting should be facilitated, and the diffusion time (time taken by the filaments to cool to glass transition temperature) should be increased. Dependence between diffusion time and process parameters was studied by Zhou, et al. (2017) [73] who used an IR sensor to obtain temperature history. Their study concluded that the increase in the nozzle and platform temperatures prolongs the diffusion time and hence this will result in increased bond strength as well as increased overall mechanical properties. However, temperatures were not as significant as printing speed which is the most dominant parameter.

**Figure 7:**
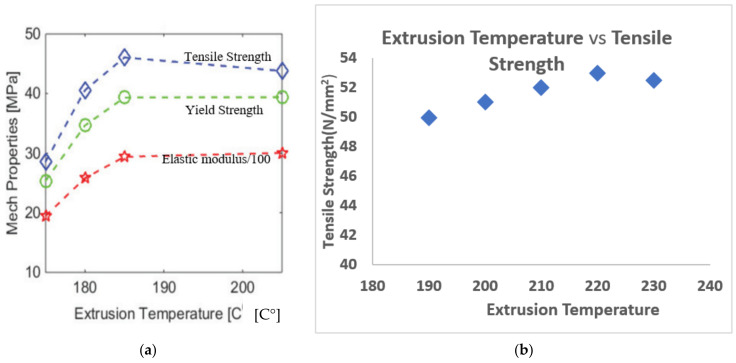
Effect of the extrusion temperature on the mechanical properties of the PLA part. (**a**) Extrusion temperature versus mechanical properties (Alafadgani, et al. (2017)), (**b**) Extrusion temperature versus ultimate tensile strenght (Jatti, et al. (2019)).

Similar conclusions were derived by Jatti et al. (2019) [60] and Alafadgani et al. (2017) [64] as shown in Figure 7. It can be seen that both researchers found that an increase in the extrusion temperature positively influences the mechanical properties of the parts. However, the increase of the UTS with the increase of temperature as overserved by Jatti et al. [60] was not as rapid as in the case of Alafadgani, et al. [64]. This might be explained by the fact that different designs of the experiment were used. The latter work involved L18 Taguchi design, while the former research varied only in the extrusion temperature while keeping all other printing parameters constant. However, by observing the trend, it can be concluded that the strength–extrusion temperature relationship is not linear, and it reaches a maximum at around 200–220 °C, above 220 °C the mechanical properties start to deteriorate. A similar trend was observed in the study undertaken by Benwood, et al. (2018) [74], in which the tensile and flexural strengths increase with the extrusion temperature until they reached a maximum and stabilized at 200 °C. Jatti, et al. (2019) [60] found that an increase in the extrusion temperature can lead to the more brittle part. Similar results were obtained by Huynh, et al. (2019). Investigations using Taguchi’s optimization of the extrusion temperature, speed and layer thickness showed that the extrusion temperature is the most significant parameter (Rank 1) that influences the mechanical properties of the part.

It should be noted that the following strength–extrusion temperature relationship is not limited to ABS and PLA materials. Ouballouch, et al. (2019) [75] studied the effect of the process parameters on the mechanical properties of the additively manufactured glass and Kelvar reinforced polyamide matrix composite parts. It was found that in both cases, the same trend was observed and the ultimate tensile strength of the parts increases with the temperature up to a certain limit.

**Figure 8:**
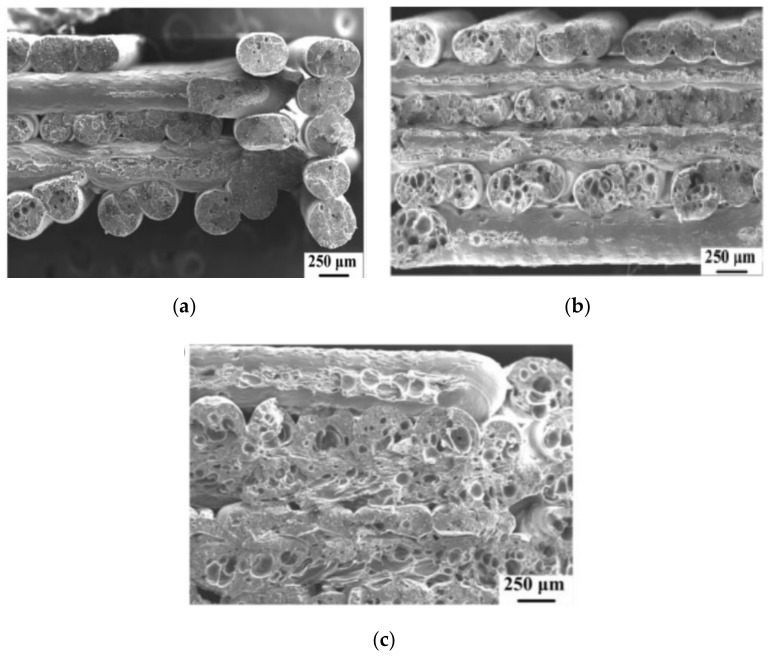
SEM images of the fracture surfaces printed at varying temperatures: (**a**) 200 °C; (**b**) 220 °C; (**c**) 240 °C [76].

The adverse effects of high extrusion temperatures were studied by Ning, et al. (2016) [76]. In their work, they used carbon fiber reinforced polymer (ABS is matrix material) and studied the effect of process parameters on the standard specimen. Fracture surfaces were then analyzed using SEM. It was found that as the extrusion temperature rises, the part becomes stronger, however after it increases above 220 °C, the mechanical properties suddenly drop. Figure 8 shows the SEM images of the fractured surfaces printed at temperatures 200–240 °C in 20 °C increment. As it was expected, the bonding between layers at low temperatures was poor (Figure 8a) and it improved up to 220 °C (Figure 8b). However, a further rise of temperature increased the fluidity of molten plastic, due to which the filaments lose their viscosity and void were constantly produced reducing the mechanical properties of the part (Figure 8c). These results were confirmed by Guessasma, et al. (2019) [77], who studied the effect of the printing temperature on the PLA-PHA plastic. The results showed that with the increase of the temperature up to 240 °C, the tensile strength and strain at break increased. However, as the temperature increased to 250 °C, the mechanical properties started to become poorer.

### 2.4. Effect of the Nozzle Diameter

The nozzle diameter also affects the mechanical properties of the parts produced by FDM. By controlling the nozzle size and layer thickness together it is possible to control the air gap between adjacent plastic strands. Its effect was studied in several works summarized below.

Kuznetsov, et al. (2018) [78], found that the strength across the printed layers (*z*-axis) is weaker than along the deposited filaments (*x*- and *y*-axis). In the first case, the strength of the bonding between the layers is a more contributing factor than the strength of deposited threads. It was found that at constant layer thickness, an increase of nozzle diameter resulted in higher flexural strength. Additionally, their results suggest that the strength increases with the layer thickness decrease. Hence, when the ratio of nozzle diameter to layer thickness increases, the contact surface between the layers increases as well resulting in higher ultimate flexural strength. This was confirmed by Vicente et al. (2020) [64] (already in the reference list), who also found an increase in the tensile strength and stiffness when the nozzle diameter was changed from 0.4 mm to 0.6 mm. This was explained, by the authors, by the gravitational force that helps to spread the melted plastic and it is more efficient for large nozzle diameters.

Triyono et al. (2020) [79] investigated the effect of nozzle diameter on the ultimate tensile stress (UTS) of 3D-printed PLA parts. The nozzle diameters used in this study varied from 0.3 to 0.6 mm. The layer thickness was set as 20% of the nozzle diameter. It was found that when the nozzle diameter is increased the UTS also increases. Using scanning electron microscope imaging, it was found that the increase in UTS was due to the reduction of the voids (air gaps) between adjusting strands. As the nozzle diameter was increased the raster becomes wider and the overlapping between neighbouring strands occurs and fused together during the solidification. This led to the reinforcement of the specimen. In this study, it was also found that the increase of UTS with the nozzle diameter occurs even if the layer thickness is increased (as the layer thinness is 20% of the nozzle diameter). This finding suggests that the ratio of the nozzle diameter to layer thickness proposed by Kuznetsov et al. is the parameter that influences the tensile strength of the part. Similar conclusions were obtained by Yang et al. (2018) using response surface methodology [80].

Nabipour and Akhoundi (2020) [81] investigated the effect of the nozzle diameter on the UTS of the ABS specimens using Taguchi-based design of experiments. It was found that for ABS material, unlike in their PLA counterparts, there is an inverse correlation between the nozzle diameter and UTS. When compared with other process parameters the contribution of the nozzle diameter to the UTS was the lowest. It must be noted that large nozzles, from 0.5 to 1.5 mm. In this study, the effect of the nozzle diameter to layer ratio was not investigated and the nozzle diameters used were larger than the ones used by Kuznetsov et al. and Triyono et al. This might be one of the possible reasons for the discrepancy between the results.

In conclusion, from the literature, it appears that the ratio of the nozzle diameter to the layer thickness may be a major factor affecting the UTS and Flexural strength of the FDM printed parts. However, it seems there are some limitations that need to be further investigated. In addition, there are currently limited data available on the effect of the nozzle diameter or nozzle diameter/layer thinness ratio, on the impact and compressive strength. Therefore, there is a need for further investigation in this field.

### 2.5. Effect of Layer Thickness

It was shown that the layer thickness also influences the strength of the printed part. On the one hand, it was found in the literature that the tensile strength of both PLA and ABS filaments decreases as the layer thickness increased. On the other hand, the impact resistance and compressive strength of parts were found to have a direct relationship with the layer thickness parameter. This means the compressive strength is improved by using larger layer thickness. This relationship might not be true for the low levels of infill density parameter as the large voids inside the parts may decrease the compressive strength. Therefore, the interaction between various process parameters should be considered depending on the loading of the part as well as specific printing conditions. Moreover, it was found that this parameter has less effect on the strength of ABS parts and is more prominent for PLA filaments.

**Figure 9:**
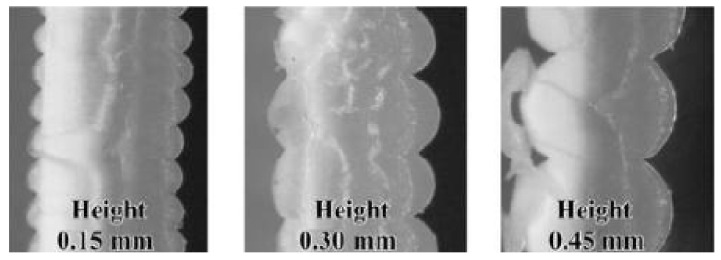
Increase in contact area with the increase in layer thickness (Coogan et al. (2016)) [25].

With increasing the layer thickness for PLA specimen subjected to tensile test, its UTS decreases (Jatti et al. (2019)) [60]. Similar results were found by Randriguez-Panes et al. (2018) [71] and Coogan et al. (2016) [25]. This might be explained by the fact that with the decrease of the layer thickness, the molten plastic extruded from the nozzle is compressed between the nozzle and platform or existing layer. Thus, instead of keeping its cylindrical shape, it deforms plastically and its cross-section becomes oval. This increases the contact area (bonding width) and wetting and therefore a fusion of the filament becomes better as shown in Figure 9 (Coogan et al. (2016)) [25].

Similar findings were obtained by Garzon-Hernandez et al. (2019) [82]. They developed coupled simulation first to find the temperature history of the deposited filament and then use this result to predict the void ratio in the cross-section. This numerical parametric investigation suggested that the increase of the layer thickness causes the decline in strength and Young’s modulus.

Alafadgani et al. (2017) [66] suggested that the increase in the layer thickness will increase the mechanical properties due to the fact that a smaller number of layers is needed.

While having a smaller layer thickness in the FDM-ed part subjected to tension is beneficial, this is not a case for compressive and impact loadings. Sharma et al. [83] found that increasing the layer thickness from 0.1 mm to 0.3 mm caused an increase of the compressive stress from 33 to 42 MPa. This was attributed to the fact that during the compression the specimen fails due to shear stress caused on the principal plane. The increased number of layers (lower layer thickness) is more prone to such failure mode. Jatti et al. [60] also found that the impact resistance of the part decreased with the reduction of the layer thickness. This finding is in agreement with the results of Ramkumar (2019) [62].

The dependence of the strength on layer thickness seems to be more difficult to establish than the extrusion temperature previously. Oubalouch et al. (2018) [65,84] investigated the effect of the layer thickness on the mechanical properties of the PLA part for different infill densities. While the combination of lower thickness–higher strength was true for infill densities of 50% and 100%, this was not true for a 10% infill density. The ultimate tensile strength increased from 30 MPa to 40 MPa when the layer height was increased from 0.2 to 0.3 mm. The underlying mechanism behind this was not provided by the authors and therefore further research on the layer thickness effect at low infill density level is needed to confirm and explain these results.

Finally, the effect of the layer height seems to be also dependent on the filament material. It was found that the ABS part was marginally sensitive to the effect of the layer thickness. Randriguez-Panes, et al. (2018) [61] compared this effect on ABS and PLA filaments and found that in the case of ABS material the effect of the layer height is insignificant, while in the case of PLA, the result agrees with the abovementioned findings. Similar results were also obtained by Samykano et al. (2019) [85]. It appears that this due to the inherent properties (chemical and mechanical) of the filament material. However, the causality of this phenomenon is still yet to be explained. Table 2 presents a tabulated summary of the finding of the effect of layer thickness on the mechanical properties of a part.

### 2.6. Effect of Raster Angle

Most of the studies primarily investigated the effect of raster angle on tensile, flexural, and impact strength of ABS printed parts. It was reported that the minimum level (0°) of raster angle improves the tensile strength of FDM parts, while the impact strength can be improved using a 45°/−45° (staggered raster) raster angle. Wang et al. (2007) [87] studied the effect of raster angle on the tensile strength of ABS printed parts using the different three levels of raster angle (0°, 45°, and 90°). The experiment was designed according to the Taguchi L18 array. The findings of the author support the previous studies showing the 0° raster angle as an optimal level. In this article, the minimum level raster angle resulted in the maximum tensile strength of 24.36 MPa. Durgun and Ertan (2014) [55] also investigated the effect of raster angle on the tensile and flexural strength of ABS parts using five levels (0°, 35°, 45°, 60°, and 90°) of the parameter. Their conclusions appear to be consistent with the previous literature reporting that the 0° raster improves the mechanical strength of FDM parts because of the larger raster length values. Nidagundi et al. (2015) [88] studied three levels of the raster angle parameter, namely 0°, 30°, and 60° using Taguchi L9 orthogonal array design and ABS resin material. The results of the SN ratio demonstrated that the ultimate tensile strength of parts decreases as the raster angle increases. Therefore, the authors concluded that the 0° raster angle is the optimal level in terms of tensile strength along with 0.1 mm layer thickness and 0° part orientation. The maximum tensile strength of 27.674 MPa was obtained using the minimum levels of three parameters as mentioned earlier.

Similarly, Panda et al. (2009) [89] and Sood et al. (2010) [13] studied the same three levels of the raster angle as Nidagundi et al. (2015). Their experiments were conducted using a central composite design and ABS plastic was used in both studies. However, the optimal level of raster angle in terms of tensile and impact strength was found to be almost 60°. Panda et al. (2009) [89] found that 54.7311° raster angle was optimal for increasing the flexural strength of parts. This was because smaller raster angles result in longer rasters that serve as the stress concentrators. This in turn causes weaker bonding and leads to poor mechanical performance.

**Figure 10:**
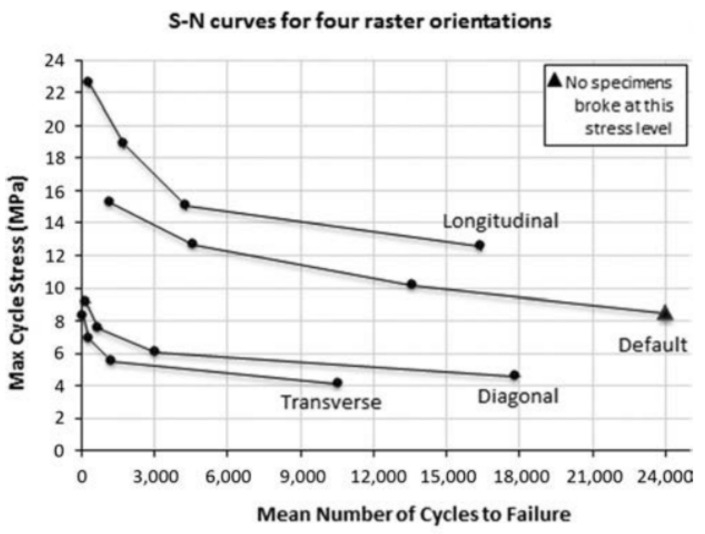
The tension–tension fatigue test results for acrylonitrile butadiene styrene (ABS) parts with different raster orientation: longitudinal (0°), default (+45°/−45°), diagonal (45°), and transverse (90°) [90].

Onwubolu and Rayegani (2014) studied the influence of the raster angle on the tensile strength of FDM printed parts using ABS resin. The two levels of the raster angle, 0° and 45°, were selected. The experiment was designed using the full factorial design and the differential evolution method was implemented to identify the optimal levels of process parameters. The main findings show that there is a direct relationship between raster angle and tensile strength of printed parts. For example, the tensile strength of parts using 0° and 45° raster angles were 32.56 MPa and 34.61 MPa, respectively. The other parameters such as layer thickness (0.127 mm), part orientation (0°), raster width (0.2032 mm), and air gap (−0.00254 mm) were kept constant. Nevertheless, it should be noted that with the increase in raster angle value there is an insignificant improvement (2.05 MPa) in the tensile strength property. Moreover, the findings of Zieman et al. (2015) [90] further support the previous literature that found 0° raster angle as the optimal level in improving the tensile behavior of ABS printed parts. The experiment was conducted using four levels of raster angle: 0°, 45°, 90°, and 45°/−45° (the latter represents the crisscross raster). The mean ultimate tensile strength was found to be a maximum of 25.15 MPa using 0° raster angle, whereas this value for 90° raster orientation was 9.16 MPa. This is because tensile strength depends on the alignment between the axis where the stress is applied and the fiber axis of printed parts. Therefore, increasing the raster angle results in a misalignment between two axes causing weaker parts in terms of tensile strength. The fatigue test result for parts printed using different raster angles is shown in Figure 10. It was found that the default setting of the raster angle parameter (45°/−45°) results in the longest mean number of cycles to failure. The second-best level of raster angle was 0° in terms of fatigue strength.

In the case of PLA resin, Liu et al. (2016) [91] found that the raster angle of 0° is optimal and results in the highest tensile and flexural strength. The authors studied three levels of raster angle such as long-raster (0°), long-short-raster (+90°/0°), and staggered-raster (+45°/−45°). The long-short-raster means layer with 90° raster angle is followed by a consecutive layer with 0° raster angle during the printing process. Based on the results of ANOVA analysis that demonstrates the percentage contribution of parameters, it was found that the raster angle parameter mostly affects the impact strength (0.127%) of PLA parts compared to tensile (0.002%) and flexural strength (0.034%). The optimal level in terms of the impact strength was found to be staggered-raster (+45°/−45°).

### 2.7. Effect of Build Orientation

The build orientation refers to the orientation of the printed parts with respect to the *z*-axis. Usually, the x-y plane is considered as the build platform area and the *z*-axis refers to the height of the printed parts. Most research indicates lower levels (0° or 15°) of build orientation to be optimal in terms of the tensile strength of FDM parts, whereas the flexural and impact strength properties show different optimal orientations in different studies. Zhou et al. (2017) also reported on the effect of build orientation as an important parameter to be impacting mechanical properties. Their study reported that the FDM part having the filaments deposited in load direction (mode II) exhibits higher tensile strength compared to the FDM part with fibres oriented in the transverse direction (mode I). The reason behind this was explained as follows. Filaments themselves can resist the load when oriented in the load direction, while filaments oriented in the transverse direction have only the bonding forces between them to resist the load. Mode III combination of mode I and mode II shows intermediate outcome [92]. Nidagundi et al. (2015) [88] reported the effect of build orientation on the ultimate tensile strength of ABS parts. The authors studied this parameter using three levels of control such as 0°, 15°, and 30°. The experimental results showed that the mean SN ratio decreases as the orientation angle increases and the larger SN ratio represented better tensile strength. This means 0° build orientation is considered as optimal in terms of the ultimate tensile strength of FDM printed parts. Apart from that, the influence of the build orientation parameter on the tensile strength of parts was reported to be 37.33% and thus being the most influential parameter compared to layer thickness and fill angle parameters. Raju et al. (2018) [93] also used SN ratio plots to study the effect of build orientation on the mechanical performance of ABS parts. On the contrary to previous studies, among the three levels (30°, 60°, and 90°) of control, 60° build orientation is found to be optimal in terms of both tensile and flexural strength of parts.

**Figure 11:**
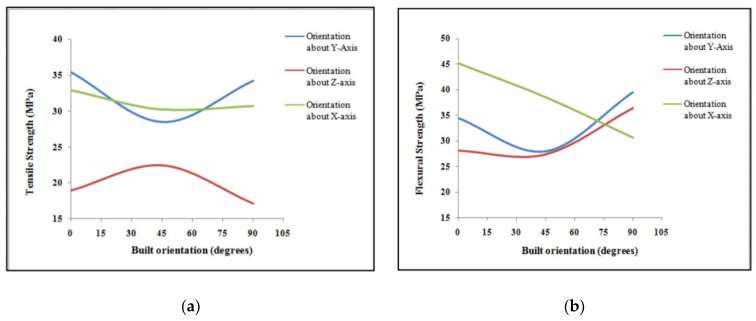
The mechanical strength of ABS printed parts using different build orientation levels: (**a**) Tensile strength; (**b**) Flexural strength [95].

Vishwas et al. (2018) [94] studied the build orientation effect using resins such as ABS and Nylon. The main findings show that the ultimate tensile strength was maximum (26.41 MPa) for ABS printed part using 0.1 mm layer thickness, 15° orientation, and 1.2 mm shell thickness. The contribution of the build orientation parameter to the tensile strength of parts was 72.41% among the three process parameters. In the case of Nylon resin, the maximum ultimate tensile strength (25.48 MPa) was attained using 30° build orientation in combination with 0.1 mm layer thickness and 1.2 mm shell thickness. Another comprehensive study of the build orientation parameter found that the lowest build orientation is optimal for the tensile strength of ABS parts (Raut et al., 2014) [95]. The authors studied the three different levels of build orientation (0°, 45°, and 90°) with respect to each of x, y, and z-axes separately. The results demonstrated that the maximum tensile strength can be observed when the build orientation parameter is set to 0°. For example, 35.45 MPa, 22.51 MPa, and 33.00 MPa tensile strength values were reported using 0° build orientation with respect x, y, and z-axes, respectively. In the case of the flexural strength, the higher build orientation levels resulted in better flexural strength values excluding the *x*-axis. The maximum flexural strength of 45.20 MPa was noted using the 0° build orientation with respect to the *x*-axis. The illustration of the relationship between the tensile and flexural strength of ABS parts and different build orientation levels with respect to x, y, and z-axes is shown in Figure 11.

Hernandez et al. (2016) [96] selected five different levels of build orientation parameter such as 0°, 45°, 90° in the XY plane and 45°, 90° in the Z plane. The mechanical strength of ABS parts was addressed in terms of tensile, compressive, and flexural strength results. The primary findings showed that the maximum tensile strength of 10.8 MPa was achieved using 90° in the XY plane. Despite this, the minimum tensile strength value was 9.36 MPa using 90° in the Z plane. This implies that the tensile strength of parts is less affected by the build orientation parameter. On the other hand, the compressive and flexural strength of parts were found to be highly dependent on this parameter. The compressive strength increased from 29.4 MPa (using 45° in the XY plane) to 59.3 MPa (using 0° in the XY plane). The maximum flexural strength of 122 MPa was also observed using 0° in the XY plane. Wang et al. (2007) [87] analyzed the effect of the build orientation parameter on the tensile strength of ABS parts using Taguchi L18 orthogonal array design and Gray theory to find an optimal process parameter. The one notable difference of this study is that the authors addressed build orientation as a categorical parameter and used the following three levels: L (the length of the printed part is the lowest in the *z*-axis), H (the length in the *z*-axis is the highest), M (the length of the parts in the *z*-axis is between L and H). Among the investigated six process parameters, only build orientation was found to have a significant impact (77.16% contribution) on the tensile strength of parts according to the ANOVA analysis. The maximum tensile strength of about 24 MPa was noted using M (medium) level of build orientation while L (low) and H (high) levels resulted in nearly 15 MPa and 14 MPa, respectively.

Abdelrhman et al. (2019) [97] investigated the build orientation using PLA resin and five levels of control as follows: X0°Y0°, X90°Y0°, X0°Y90°, X0°Y45°, and X90°Y45°. The tensile strength and maximum fracture load of printed parts were considered as the output of the experiment. Both the maximum average tensile strength of 29.36 MPa and fracture load of 1409.09 N were achieved using X0°Y0° build orientation. It was noted that the mechanical behavior of PLA parts worsens as the Y-component of build orientation increases. For example, the minimum average tensile strength (14.71 MPa) was noted using X0°Y90°. Liu et al. (2016) also studied the effect of build orientation on PLA printed parts using the following levels of control: 0°, 60°, and 90°. The results of the Taguchi L27 design experiment showed that 0° build orientation is the optimal level in terms of tensile, flexural, and impact strength of FDM parts. This was confirmed by implementing grey relation analysis. The maximum tensile (50.34 MPa), flexural (83.51 MPa), and impact (23.07 MPa) strength values were obtained using the following optimal combination of process parameters: 0° build orientation, 0.3 mm layer thickness, 0° raster angle, 0.5 mm raster width, and −0.1 mm raster gap.

## 3. Impact of Pre-Processing on the Strength of FDM

Fused deposition modeling (FDM) as any other technologies has limitations such as low structural strength and surface finish (stair-stepping effect) of the parts. Although manipulation of process parameters makes significant differences in the quality and mechanical properties of the printed part, the ideal combination of parameters is almost impossible to achieve (Popescu, 2018) [7]. According to Hongbin Li et al. (2016) [3] infill rate, deposition velocity and layer thickness highly affect the result of the manufacturing process. Enhancing one parameter usually sacrifices the other. For example, to increase interlayer bonding the layer thickness must be decreased, thus resulting in a longer printing time [3] (Li, 2016).

One of the main concerns arising is due to anisotropic behavior of the extruded layers and the part in general. This issue is generally attributed to poor inter-layer bonding. One of the solutions for increasing the mechanical properties of the printed parts without sacrificing the other parameters is preheating of the extruded layer. The principle is to preheat the extruded layer (existing later) before the next layer is deposited. This can be ensured by different kinds of light sources such as laser or infrared light for example. The preheating of the surface of the already extruded layer up to the glass temperature in order to increase the bonding between layers was proposed by Kishore et al. (2017) [98] (Kishore, 2017). Several methods of preheating with various light sources, materials and printing parameters have been investigated. Each method has been studied and analyzed. A comparison table (see Table 3) summarizes the different techniques.

Kishore et al. (2017) developed one of the approaches that has been proposed for enhancing the quality of the printed parts. The methodology of this experiment was applied for big area additive manufacturing systems (build volume 6 m × 2.5 m × 1.8 m) with an infrared lamp as a light source. Material that was used during this experiment—fiber reinforced thermoplastic pellets (acrylonitrile butadiene styrene (ABS) reinforced with 20 wt.% short carbon fiber) instead of filament to optimize the cost [98] (Duty, 2017).

**Figure 12:**
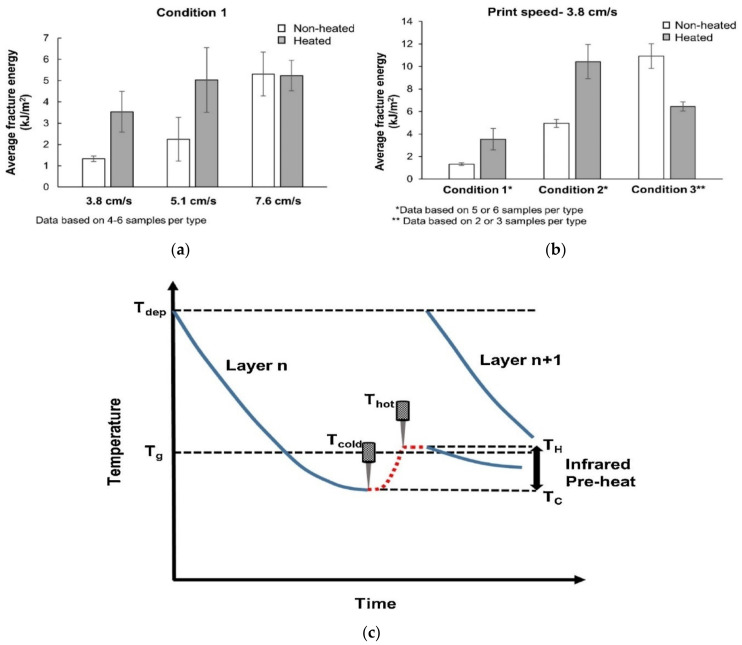
(**a**) Variation of fracture energy with print speed (**b**) Variation of fracture energy for various pre-heating conditions (**c**) Schematic of time–temperature profile. (Kishore, 2017) [98].

The effect of lamp power was investigated by three different experiments where the position and intensity of Infrared lams varied. For the first condition, print speed was varied (3.8 cm/s, 5.1 cm/s, 7.6 cm/s) and two 50 W IR lamps were arranged at an angle to maintain the printed surface 8 cm away from the lamps. Figure 12a shows the results of the experiment. At 3.8 cm/s the result of the average fracture energy becomes more than doubled after heating the layer. A similar result was achieved at the second speed variation; however, the last run reported a slight decrease in the average fracture energy after preheating compared to the initial value for the non-heated run. For the first two cases, the printing speeds were relatively low, therefore more cooling of the surface was compensated by preheating, nevertheless, the speed was higher for the third case, and hence the plastic did not have time for overcooling, thus improving the “non-heated” results.

At this time, condition 2 had the same IR lamps but placed at a distance of 2.5 cm away whereas condition 3 used 1 kw IR lamp (single 6.35 cm long) placed at a distance of 1 cm above the deposited bead.

The comparison of three conditions with varying heating arrangements but with the same speed setup (3.8 cm/s) was also reported in Figure 12b. Preheating for the first and the second condition increased the average fracture energy more than twice. However, the third condition showed poor results, due to the small distance between the light source and layers which led to the plastic degradation and eventual reduction in tensile strength in the z direction [99]. One of the problems faced during these experimentations was the inability to preheat the extruded layer to the desired glass transition temperature. Figure 12c demonstrates the temperature profile with preheating by IR lamp. Large part usually suffers from long layer time and therefore experiences cooling of the layer below glass transition temperature from the deposition temperature. With the incorporation of IR lamps, substrates temperature can be elevated closer to the glass transition temperature just before the next layer deposition and thus enhances the interlayer bonding strength. However, the competency of the preheating largely depends on the lamp intensity as well as lamp orientation, standoff distance, and printing speed as well. On the other hand cooling rate of substrates depends on ambient temperature, geometry variation, tool path pattern and thermal conductivity of the materials [100].

In order to avoid the excessive heating experienced by some preheating conditions previously, Kishore et al. (2019) [99] also developed an artificial cooling system to cool down the extruded layer before exposing it to the IR light. The temperature was decreased by forced convection down to 40°C, 60°C and 80°C with the variation of the active cooling cycle. The idea was to always start heating the surface from the set temperature in order to achieve 150 °C. This technique prevented plastic from overheating, thus reducing the risk of plastic degradation. The average tensile strength in z-direction was increased by 81% for T_cold_ = 40°C.

**Figure 13:**
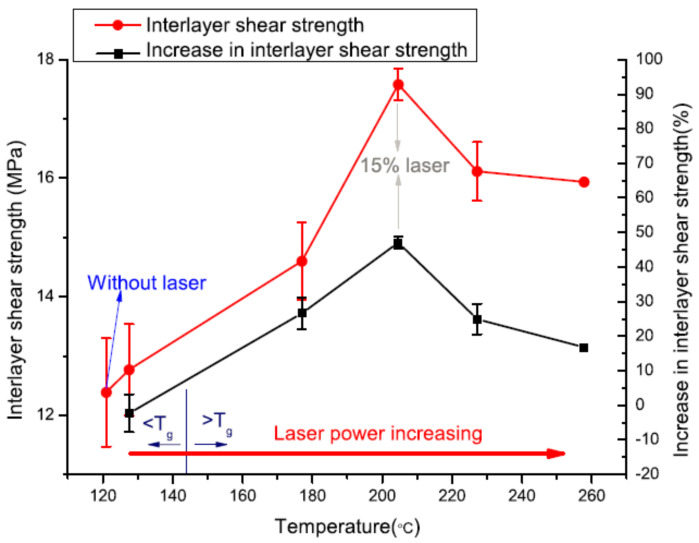
Interlayer shear strength and percentage increase of interlayer shear strength with different interlayer bonding point temperatures (Luo, 2018) [101].

Another approach of enhancing FDM printing was developed by Meng Luo. It was based on “laser assistance”. The CO_2_ laser device (40 W) was implemented into a traditional FDM printer. The setup required additional mirrors to locate the laser beam on the extrusion path. Control of the mirrors makes the entire system more complex. (Luo, 2018) [101]. Interlayer shear strength was directly affected by the preheating as shown in Figure 13. Preheating results in a small enhancement in interlayer bonding for the temperature of the laser point less than glass transition temperature. However, temperature over glass transition temperature results in significant improvement. Interlayer shear strength polyether ether ketone (PEEK) was improved up to 45% however, other laser power variations resulted in poor outcomes. The optimal laser power should be 15% of the full power. For the complete understanding of the effect of the parameters on the sample’s properties, parameters such as laser power, laser focusing angle and printing speed are needed to be analyzed further.

**Figure 14:**
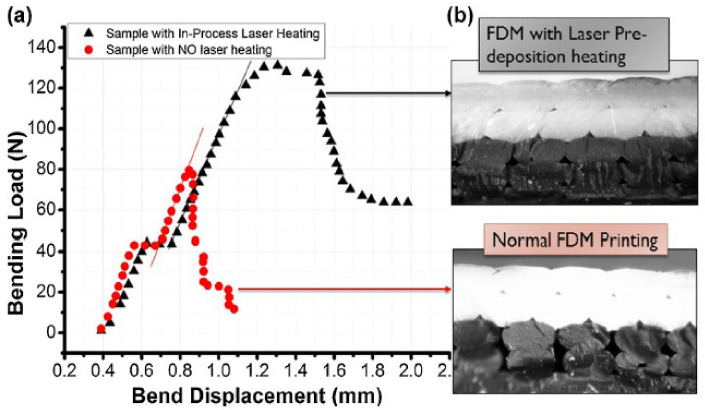
(**a**) Bending load–deflection plot (**b**) Optical micrograph of freeze-fractured control samples and those using the in-process laser pre-deposition heating approach (Ravi et al., 2016) [102].

Ravi et.al (2016) also implemented preheating apparatus for obtaining better results in terms of isotropy. Equipment was set up based on the commercial open-source 3D printer (Ravi, 2016) [102]. A combination of the laser and optic system was used as a light source (laser with the power of 2 W). Installation of the mirrors was added on the platform. It was determined that at lower printing speeds, the laser provides an excessive amount of energy to the plastic, thus causing degradation and defects. In order to analyze the effect of preheating on flexural strength, bending tests were performed. The design proposed by these researchers showed a 50% increase in the strength of the interlayer bonding. In addition to that, the ductile behavior of the plastic after preheating was also noted. As can be seen from Figure 14a, a sample without laser preheating experiences brittle fracture at the point where a sharp drop in bending load occurs at the end of the linear load–deflection curve, whereas for preheated sample, failure occurs in a ductile manner at the end of the linear load–deflection curve due to the increased bond potential as raised by increased temperature as well as interpenetrating diffusion. In addition, optical images in Figure 14b show the clear improvement of bonding strength with respect to nonheated FDM printed sample. In the case of the laser-treated sample, a more uniform profile suggests the reflow of substrate material due to heating which promotes the diffusion mechanism and therefore reduces defect between the layers subsequently.

**Figure 15:**
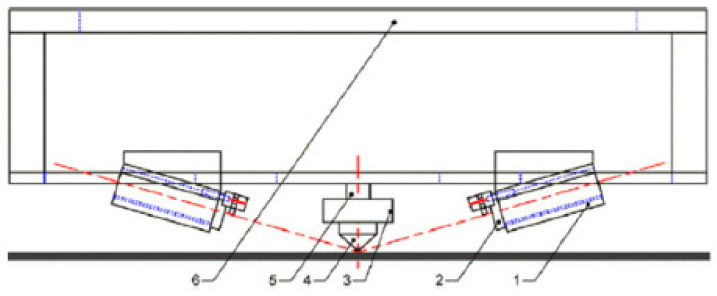
Experimental setup (1) fixing equipment; (2) infrared laser; (3) nozzle heater; (4) nozzle; (5) filament source; (6) frame (Du, 2016) [103].

Du et al. (2016) [103] deployed two laser sources for preheating, post-heating (Figure 15) or lateral heating in case of large thin-walled part fabrication. Two-directional heating is the main advantage of this setup. Results of this approach have been generated by simulations based on governing equations. Semi-implicit pressure-linked equation algorithm was implemented to simulate FDM printing. The flow of the melted plastic was taken into account by Navier–Stokes and energy equations (Du, 2016). Laminar incompressible flow assumptions were also made. A 195% increase in the tensile strength was achieved by the lateral laser-assisted heating. For the same printing speed and laser power, lateral heating is reported to be more effective in achieving higher bonding strength compared to pre- and post-heating. Effective bonding width was reported to be increased by 24% using pre- and post-laser heating. The optimal value of speed ratio (wire feed rate vs printing speed) was determined to be 0.75. The optimal value of the layer thickness was found to be 0.25 mm (Du, 2016) [103].

**Figure 16:**
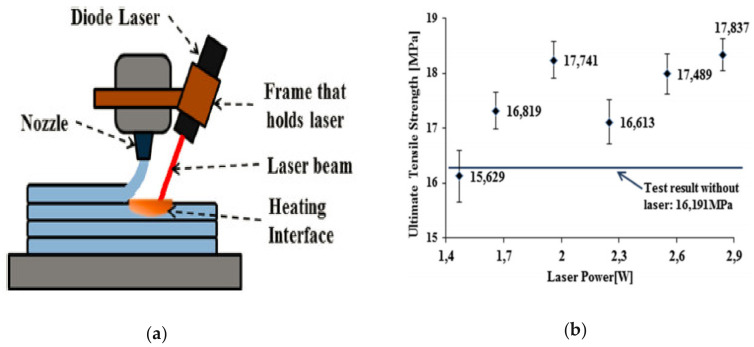
(**a**) Equipment setup (**b**) Ultimate stress vs. laser power graph [104].

Sabyrov et al. (2020) also proposed the application of a 5 W diode laser for preheating the surface of the extruded layer due to the waste of power involved in heating big areas of the layers. Their setup does not require additional components such as mirrors as shown in Figure 16a. Additionally, the setup proposed by Kishor et al. (2019) is not applicable to common FDM equipment. Sabyrov et al. (2020) reported a 10.16% increase in ultimate tensile strength at a 2.84 W power laser (Figure 16b). However, the quality of the parts seems to be very poor. Cracks and holes were evident in the part due to intensive laser heating. Since the result associated with 1.66 W and 2.84 W laser are the best and are not very different from each other, Sabyrov et al. (2020) suggested using a 1.66 W laser to reduce energy consumption. In addition, the color of the plastic can also influence the performance as darker material absorbs more energy compared to light color materials. Further investigation is needed in the following areas: printing speed, direction angle, size of the focal point of the laser, laser diode-based heating on the bending parameter [104].

**Table 3:** Summary of Enhancement techniques.

Articles	Material	Printing Speed (cm/s)	Deposition Temperature	Light Source	Power of the Light Source	Preheating Temperature
(Kishore et al., 2017) [98]	Acrylonitrile butadiene styrene (ABS) reinforced with 20 wt.% short carbon fiber	3.8, 5.1, 7.6	215 °C	Infrared lamp 500 W for case 1 and 2 1 kW for case 3	100% for case 1 and 2 80–90% for case 3	N/A
(Kishore et al., 2019) [99]	Acrylonitrile butadiene styrene (ABS) reinforced with 20 wt.% short carbon fiber	5.1	215 °C	Strip IR model number 5306B-02-1000-01-00 (the same lamp that was used for case 3 previously)	80%	150 °C
(Luo et al., 2018) [101]	semicrystalline thermoplastic polymers	0.6	410 °C	40 W CO_2_ laser (10.6 μm wavelength)	5, 10, 15, 20, 25%	Varied with the power of the light source
(Ravi, 2016) [102]	black-color ABS filaments	0.1–1	230 °C	802 nm solid-state laser (2 W)	0.75 W	Varied with the power of the light source
(Du, 2016) [103]	ABS polymers	1–2	Not specified	Laser (2 W)	0–2 W	By 20–30 °C
(Sabyrov, 2020) [104]	PLA plastic	3.5	210 °C	Diode laser (5 W)	1.47, 1.66, 1.96, 2.25, 2.55, 2.84 W	Varied with the power of the light source

Since warpage and porosity of printed part are impacted by environmental temperature and humidity, some research suggested to use heated printing chamber to improve the FDM printed part quality. Fang et al. (2020) investigated the impact of environmental temperature and reported that temperatures from 30 °C to 90 °C could potentially improve the mechanical properties along printing direction due to reduced warping defect [105]. Sun et al. additionally experimented with the impact of built chamber temperature on polyether ether ketone (PEEK) polymer and reported profound improvement in the printed part strength [106]. Sproerk et al. (2018) also reported a similar finding and suggested the occurrence of annealing with reduced warpage of printed part as a result of enhanced printing chamber temperature [107,108]. Carneiro et al.(2019) [109] and Armillotta et al. [110] also suggested the reduced structural porosity due to increased chamber temperature close to the glass transition temperature.

It is also evident that mechanical properties are negatively influenced by the presence of pores in the structure which helps crack propagation [111]. Since the percentage and volume of pores increase with the increased water content, the mechanical strength of the printed part decreases in both longitudinal and transverse loading directions in the presence of moisture content, however, some improved ductility may be observed [112]. Moisture content also reduces the glass transition temperature. Fang et al. (2020) in their work suggested not only the prior drying of the filament but also to maintain a dry heated environment during printing (below 30% RH) for better mechanical properties. Their result suggested 30% reduced strength in the longitudinal direction and 70% reduced strength in transverse directions with filament with moisture content with respect to dried filaments [105].

## 4. Research Trends

In this section, the current research trends will be discussed such a vacuum-assisted FDM, advances in materials, and new technologies.

### 4.1. Vacuum-Assisted FDM

Maidin et al. (2017a, 2017b, 2018) [113,114,115] have shown that vacuum in the printing chamber improved the quality of the printing due to the decrease of heat transfer from the nozzle and hence providing a smooth and slow decrease of the temperature of the polymer that improved the bonding between the layers. The pressure in the chamber was gradually reduced from 30 inHg (or 760 mmHg of normal atmospheric pressure, however, the authors used inches of Hg) to 21 inHg (533.4 mmHg). At this pressure, the surface quality of the printed part improved compared to the atmospheric conditions, i.e., the waviness, stringing and blobs were eliminated and the surface roughness decreased by 9%. For the tensile strength, the pressure was decreased further to 18 inHg (457.2 mmHg) and it was observed that the tensile strength increased by 12.83% (from 12.85 MPa to 14.50 MPa) compared to the normal printing conditions.

### 4.2. Advances in Materials

Materials that are typically used in FDM are ABS (Acrylonitrile Butadiene Styrene), PC (Polycarbonate) and PLA (Polylactic Acid) or the combination of two such as ABS+PC (Tambrallimath et al., 2019) [116]. One of the current trends in Additive Manufacturing (AM) is the reinforcement of these polymers to enhance the mechanical properties of the printed workpieces such as tensile strength and Young’s modulus. For example, carbon fibers (Ning et al., 2015; Shofner et al., 2003 and Tekinalp et al., 2014) [117,118,119], carbon nanotubes (Dul et al., 2018 and Sezer and Eren, 2019) [120,121], ceramic particles such as ZnO (Aw et al., 2017 and Torrado et al., 2015) [122,123], graphene (Dul et al., 2018) [120] have been added to ABS to modify its mechanical properties (a summary of these investigations available in Table 3). It was shown that the new resultant properties depend on the weight percentage (wt.%) of the additives in the polymeric matrix and that there is a limit to the amount of additives that can be added to the matrix, i.e., there is an optimum value of maximum increase of desired properties. Above this percentage, the values of mechanical properties start to decrease or fluctuate. However, there are cases when properties may not increase till the maximum point and exhibit a negative effect compared to pure polymer. For instance, Torrado et al. (2015) [123] reported that the addition of nanorods made from ZnO resulted in a decrease of tensile strength due to the formation of microvoids, though the addition of exactly 2 wt.% was evaluated, i.e., the authors did not consider values that are less or more than 2 wt.% which could have given different results. Nevertheless, Aw et al. (2017) [122] reported the positive effect on tensile strength of ZnO particle addition at 11 wt.% and following that decrease of tensile strength at 14 wt.%. Another limitation reported by Sezer and Eren (2019) [121] and Tekinalp et al. (2014) [118] is the nozzle clogging that was explained to be the consequence of the additives’ agglomerations that are comparable to the diameter of the nozzle in size. Moreover, Ning et al. (2015) [119], Sezer and Eren (2019) [121], Snofter et al. (2003) [117] showed that the reinforcement leads to the enhanced brittle behavior of the composite and decreased ductility. Additionally, the sources provided information on how the feedstock filaments were manufactured highlighting the importance of the mixing procedure to insure a uniform dispersion of the additives. Zhou et al. (2020) also reported on profound improvement on the ductility of FDM part with the help of compatibilizer and nanoparticles when processing parameters are optimized. Their study stated improved tensile strain of FDM part compared to injection molded part due to the balanced relationship between bonding properties and ductility originated from adjusted porosity, and compatibility [124].

Singh et al. additionally reported on the development of filament made of high-density polyethylene (HDPE) with embedded cenospheres which contribute to lower composite density. This kind of filament with lower density can offer enhanced compressive properties [125,126]. In another study, Patil et al. additionally investigated the compressive characteristics of fly ash cenosphere-based three-phase synthetic foam printed part and demonstrated enhanced specific compressive modulus and yield strength. This kind of printed part has potential application in lightweight applications such as sub 4000 m range buoyancy modules [127].

**Figure 17:**
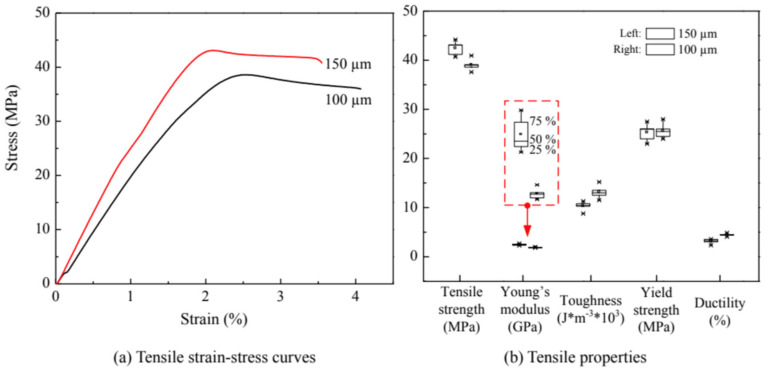
Comparison of the printed parts with the addition of 100 and 150 μm long fibers [119].

Factors that affect the results are not limited to the amount of additive only. The geometry (such as length) of the additives, their orientation in the matrix and the printing conditions are also significant factors. For instance, Ning et al. (2015) [119] compared two types of specimens that were reinforced with 150 μm and 100 μm long fibers respectively and resulting graphs showed that the addition of longer fibers produces stronger printed parts in terms of tensile strength and Young’s modulus as can be seen from Figure 17. However, ductility and toughness of 100 μm long fibers results shows better results compared to 150 μm. Tekinalp et al. (2014) [118] performed similar experiments with 200 μm and 400 μm long fibers. They also confirmed that the decrease in fibers’ length leads to a less prominent effect on tensile strength enhancement due to the reinforcement. The values of tensile strength and Young’s modulus reported in the study of Tekinalp et al. (2014) [118] were larger compared to Ning et al. (2015), however, the percentages added were different: 30 wt.% and 5 wt.% respectively. Another comparison can be drawn from multi-wall carbon nanotubes (MWCNT) studied by Dul et al. (2018) [120] and Sezer and Eren (2019) [121]. Though the dimensions of the nanotubes were the same, maximum values of Young’s modulus were achieved at different concentrations of 8 and 10 wt.% respectively and these values of Young’s modulus are drastically different. Sezer and Eren (2019) [121] reported about the increase of Young’s modulus to the value of approximately 1980 MPa (66.8% increase compared to the pure ABS), while Dul et al. (2018) [120] showed the corresponding value of 2650 MPa (19% increase compared to the pure ABS). The reason behind this is not clear but it can be concluded that other factors might have affected the results such as different manufacturers of ABS polymer, shape of the specimens and printing conditions.

**Figure 18:**
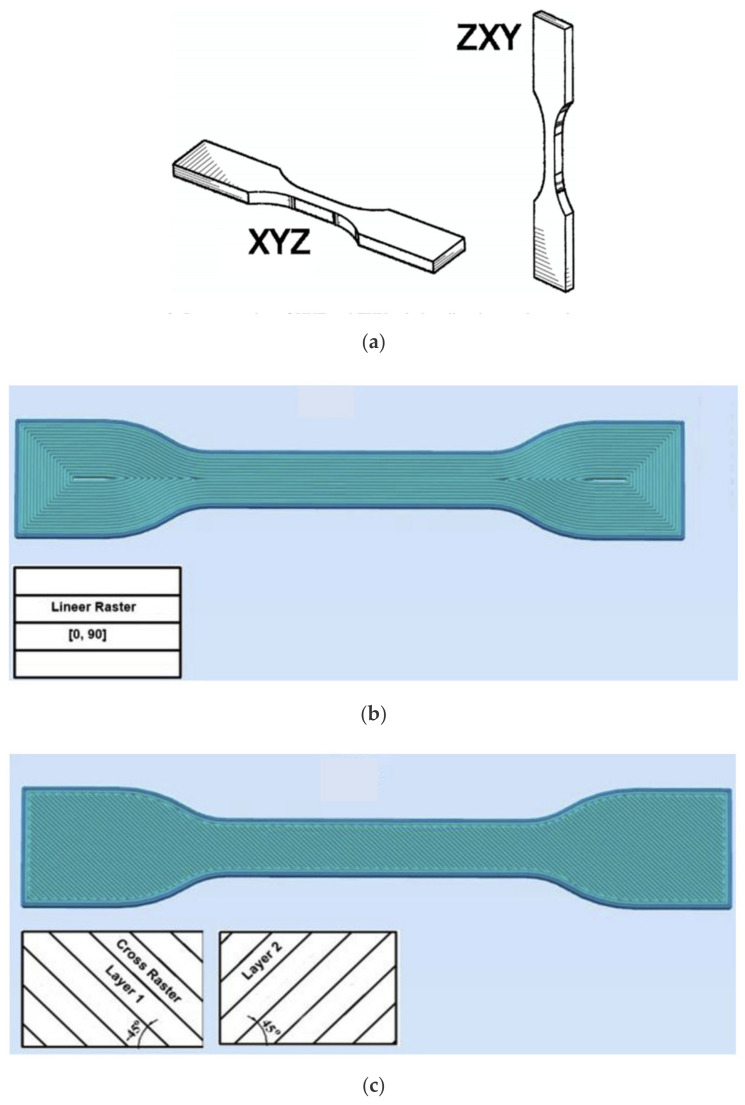
(**a**) XYZ—horizontal orientation; ZXY—vertical orientation (Torrado et al., 2015) [123] (**b**) raster angle of [0, 90]; (**c**) raster angle of [−45, 45] (Sezer and Eren, 2019) [121].

With regards to the orientation of the fibers, Snofner et al. (2003) and Tekinalp et al. (2014) reported the fact that most of the fibers tend to orient along the direction of the extrusion. According to Tekinalp et al. (2014), this is because, in FDM printing, the material flows in a specific direction through the nozzle, compared to compression molding, where the material is free to flow in any direction to fill the mold. This inherent preferential orientation resulted in an increased property since this direction of extrusion coincided with the load-bearing direction of the specimens. The extrusion can also be performed horizontally and vertically (Figure 18a) as well as at different raster angles (Figure 18b,c). According to Torrado et al. (2015) [123] and Dul et al. (2016) [128], the vertical orientation appears to be less beneficial to print with the composite material because the effect of the reinforcement on mechanical properties is less prominent. For instance, Young’s modulus increased from 1866 to 2463 MPa when specimens were printed horizontally and increased from 1687 to 2151 MPa when specimens were printed vertically (Dul et al., 2018) [120]. Moreover, Sezer and Eren (2019) [121] demonstrated the importance of the angle at which the filaments are extruded. Raster angles of 0, and 90° showed higher results (mechanical properties’ improvement) due to the orientation of the extruded filaments in the direction of the loading as shown in Figure 18b. Table 4 shows the summary of the study carried out regarding the advancement of materials in FDM.

Recently, the FDM part made of thermoplastic elastomer (TPE) also awakened deep interest due to its wide range of applications. TPE basically combines the dynamic processing properties of thermoplastic with the softness and flexibility of elastomers. Antonia et al. (2020) reported on the application of thermoplastic elastomer thermoplastic polyurethane (TPU)) filament along with conductive fillers for FDM printed strain sensors and suggested various piezoresistive responses for different filament diameters [129]. Harynska et al. (2018) reported about the development of medical-grade thermoplastic polyurethane (S-TPU) which demonstrated promising results in terms of mechanical properties as well as biocompatibility [130]. Tsai et al. (2017) also demonstrated FDM printing of tubular construct using TPU and suggested the possibility of postprocessing to include bioactive molecules [131]. Jung et al. (2016) also investigated TPU printed tracheal scaffold for an animal study and reported on sufficient mechanical strength along with high elastic properties [132]. FDM techniques when combined with elastomeric filament can lead to lots more future opportunities due to product customization and cost-efficient design.

**Table 4:** Additive composite materials for FDM.

Material	Authors	Percentage Added and Description of the Additives	Notes (The Increase/Decrease of the Mechanical Properties are Considered Relative to Pure ABS)
ABS + carbon fibers	Ning et al. (2015) [119]	3, 5, 7.5, 10, 15 wt.%; 150 μm and 100 μm in length with a common diameter of 7.2 μm	150 μm: Maximum tensile strength value—42 MPa at 5 wt.% 150 μm: Maximum Young’s modulus value—2.5 GPa at 7.5 wt.% Results of 150μm long fibers’ addition are higher than of 100μm long fibers
	Tekinalp et al. (2014) [118]	10, 20, 30, 40 wt.%; the fibers’ length—200–400 μm.	40 wt.% resulted in the clogging of the nozzle (thus the author disregarded the results of the specimens made with 40 wt.%). Orientation of the fibers was along the load-bearing direction The increase of tensile strength was from 30 MPa to approximately 60 MPa at 30 wt.% The increase of tensile modulus was from 2 GPa to approximately 13 GPa at 30 wt.%
	Shofner et al. (2003) [117]	10 wt.% only; the average diameter—100 nm, length—100 μm	Tensile strength increased by 39% (from 26.9 MPa to 34.7 MPa) Tensile modulus increased by 60% (from 0.49 GPa to 0.79 GPa)
ABS + carbon nanotubes (CNT)	Sezer, H.K., and Eren, O. (2019) [121]	1, 3, 5, 7, 10 wt.%; average diameter—9.5 nm; average length—1.5 μm; surface area—250–300 m^2^/g	Tensile strength increased by 28.8% (up to 58 MPa) at 7 wt.% and raster angle [0, 90] Young’s modulus increased by 66.8% (up to approximately 1980 MPa) at 10 wt.% and raster angle [0, 90] Specimens printed at raster angle of [0, 90] performed better in mechanical properties testing than at [−45, 45]
	Dul et al. (2018) [120]	1, 2, 4, 6, 8 wt %; average diameter—9.5 nm; average length—1.5 μm; surface area—250–300 m^2^/g	Elastic Modulus increased by 19% (approximately from 2207 MPa to 2650 MPa) at 8 wt.% Tensile strength was not provided buy yield strength increased from 42.8 MPa to 47.1 MPa at 6 wt.%
ABS + ZnO	Aw et al. (2017) [122]	8, 11, 14 wt.%; Particle’s size < 100 μm	Tensile strength increased from about 10 to 16 MPa at 11 wt.%
	Torrado et al. (2015) [123]	2 wt.% only; ZnO nanorods were used (no information about the size)	Tensile strength decreased from 33.96 MPa to 20.7 MPa when specimen was printed horizontally. Tensile strength decreased from 17.73 MPa to 7.41 MPa when specimen was printed vertically
DulABS + graphene	Dul et al. (2016) [128]	2, 4, 8 wt.%; average lateral dimension –5 μm, thickness—6–8 nm, the surface area—120–150 m^2^/g.	MFI (Melt Flow Index) was studied, and it was revealed that specimens with 8 wt.% had low MFI, hence the authors disregarded the results of 8 wt.% Tensile strength decreased from 38.8 to 35.9 MPa at 4 wt.% when specimens were printed horizontally. Tensile strength decreased from 23.8 to 13.4 MPa at 4 wt.% when specimens were printed vertically. Young’s modulus increased from 1866 to 2463 MPa at 4 wt.% when specimens were printed horizontally. Young’s modulus increased from 1687 to 2151 MPa at 4 wt.% when specimens were printed vertically.
PC + ABS + graphene	Tambrallimath et al. (2019) [116]	0.2, 0.4, 0.6, 0.8 wt.%; PC:ABS = 70:30; No dimensions of graphene platelets were provided	Young’s modulus increased from 2531 to 4038 MPa at 0.8 wt.% Tensile strength was not studied
ABS + OMMT	Weng et al. (2016) [133]	1, 3, 5 wt.%; No dimensions of OMMT nanoparticles were given	OMMT stands for organic modified montmorillonite. Montmorillonite belongs to the group of phyllosilicates. Tensile strength increased from 27.59 MPa to 39.48 MPa at 5 wt.% Elastic modulus increased from 1.2 GPa to 3.6 GPa at 5 wt.%
ABS + BAK + Al_2_O_3_ + SiC	Singh et al. (2019a) [125]	BAK: fixed value of 10 wt.%; Al_2_O_3_: 0, 5, 10 wt.%; SiC: 0, 5, 10 wt.%; No dimensions were given	BAK stands for bakelite. It is a thermoset and was used as a filler in ABS matrix for recycling purposes. For one specimen Al_2_O_3_ and SiC were added in the same quantity. For example, 70% of ABS + 10% of BAK + 10% of Al_2_O_3_ + 10% of SiC Maximum strength at peak was 24 MPa at composition of 90%(ABS) + 10%(BAK)
	Singh et al. (2019b) [126]		The specimens were studied at different infill ratios and infill speeds. Maximum tensile strength of reinforced material was slightly less than that of pure ABS: 21.8 and 22.4 MPa respectively. Maximum tensile strength was observed at composition of 70%(ABS) + 10%(BAK) + 10%(Al_2_O_3_) + 10%(SiC); infill ratio of 80 and infill speed of 50 mm/s

### 4.3. Advances in Technology

In this section, the patents were presented to show recent advances in technology. The main driving force for advances in FDM is increasing the accuracy of the printers and increasing the share market of FDM printers among inexperienced consumers. According to Shi et al. (2014) [134], the patents can be divided into three categories: (1) filament storage and printer heads, (2) support materials and (3) auxiliary measures. Selected patents are described in more detail in Table 5. In the case of a storage and management system of the filaments, according to the design, two different storage containers for modelling and support structures respectively can be integrated with the circuit board to provide information to the printing system about the amount and type of filaments left. The two storages have different housings such that they cannot be misplaced with each other by the user (Taatjes et al., 2012, US8157202) [135]. Another system was designed to diminish the shaking of the printer head in the x, y direction by installing the head mount for retaining the print head. The head mount allows the deposition head to tilt or move upward in case it is pushed by a platform below reducing its risk to be damaged (Comb et al., 2012, US8153182) [136]. Another protective patent is a safety system located near the heating element and can sense and control its temperature independently from the main controller such that the temperature will be lowered if it surpasses the optimum safe value or the value that was pre-set by the user via the main controller (Pax and Schmehl, 2014, US20140044823) [137]. With regards to the improvement of control speed during printing, there is a printer that incorporates two deposition heads (one for modelling and another one for support material) in one carriage with two receptacles. The design allows one of the heads to be lowered to print and another to stay inactively retained in the upper position until it is required to print. The relative height between two printer heads is about 0.5 mm. The mechanism allows the two heads to exchange their positions from lower to upper state simultaneously (Swanson et al., 2013, US8465111) [138]. The patents for composite filaments involve the nozzle that heats the filament and has a special ironing lip at the tip that “irons”, i.e., compacts the extruded material applying the pressure on it such that its circular cross-section becomes more rectangular resulting in stronger printed parts [139,140]. Another patent for composite filament was designed to control high-aspect-ratio fibers by rotation of the nozzle The higher the rotational speed the more perpendicular to the direction of extrusion the fibers are (Lewis et al., 2016, US20160346997) [141].

There are also patents to ease the process of removing building supports after printing. For instance, it has been shown that the addition of the carboxyl and phenyl monomers into the filament’s material can make it soluble in alkaline aqueous solution and less susceptible to cracking (Hopkins et al., 2012, US8246888) [142]. While Tafoya developed tanks that can be used to remove the support material by providing appropriate environmental conditions for the dissolution of the supports (2013, US8505560) [143].

Regarding the FDM enhancement techniques, there are certain aspects that can be improved or further analyzed. It is important to note that different plastics were used for those experiments. Therefore, it is not fully clear how beneficial each method for different materials. Another area of improvement is limited heating direction, which highly affects printing time and thus cost. Ultraviolet light sources heat up too much surface area of the extruded layer, which negatively affects plastics. A more precise method is the implementation of the lasers, however, these setups are very complex and require additional optical systems. Preheating temperature control should be studied more, since the temperature can be varied only by the power of the light source, assuming that the printing speed is constant. The power of the source depends on the size of the focal point, the distance between the light source and surface.

**Table 5:** Patents on FDM technology.

Type	Authors, Number of Patent	Bullet Points
Filament storage and printer heads	Mark et al. (2017) US9815268 [140]	The ironing lip of the nozzle that, when in contact with melted material, “irons” it onto the layers of the part, i.e., presses it as the nozzle or plate are moving relative to each other. Hence, flat compressed cross-sections of the initially circular extruded composite are created. The resultant shapes can be regulated by the controller that lowers the nozzle by 1/2 or 1/3 initially circular extruded filament’s diameter
	Mark and Gozdz (2015) US9126367 [139]	The patent was built specifically for the composite filaments to be printed by FDM The temperature in the nozzle is higher than melting temperature of the polymeric matrix and lower than melting temperature of the added fibers The fibers are presented in the paper to be continuous or semi-continuous
	Pax and Schmehl (2014) US20140044823 [137]	The heater has an integrated safety system that can regulate the temperature of the heater and maintain the safe operating temperature of the extruder
	Swanson et al. (2013) US8465111 [138]	The print heads are supposed to be loaded into the receptacles There is a voice coil mechanism below the receptacle that is controlled to be moved along *z*-axis such that the head is in the active state when lowered and in the passive state (no filament extrusion) when in the upper position.
	Taatjes et al. (2012) US8157202 [135]	The container for the filaments has a circuit board required for the communication between the printer and the container providing information about the type and amount of the filament in the container.
	Comb et al. (2012) US8153182 [136]	The deposition head is supposed to be installed into the head mount An adjustable head mount is designed to prevent damage to the printer head and secures from undesirable shaking
Support materials	Hopkins et al. (2012) US8246888 [142]	Carboxyl and phenyl monomers are added to the support material making it soluble in the aqueous alkaline solution (ph7–13) It is also claimed that the material becomes less susceptible to cracking
	Tafoya (2013) US8505560 [143]	A tank with the aqueous solution to remove the support material The temperature control is integrated to provide required conditions for material removal
Novel technique	Lewis et al. (2016) US20160346997 [141]	The rotation of the deposition head can control the alignment of the fibers (or other high-aspect-ratio additives) in the material The higher the rotational velocity the more inclined to the direction of the extrusion the fibers are.
Auxiliary measures	Paul and Batchelder (2012) US8222908 [144]	Capacitive detector that induces an electric field is used When the extruded material goes through the electric field the capacitance value rises The detected capacitance value is dependent on the volume, diameter of the extruded filament and the moisture content in it. Hence the concentration of the moisture and lack of material (due to nozzle clogging and other issues) can be detected
Biotechnology	Boehm et al. (2013) US20130326878 [145]	Production of dental prosthesis using FDM

## 5. Summary

Based on this literature review, although it may be expected that FDM process parameters should be well established (as numerous users are using this technology to manufacture parts), for some process parameters, non-conclusive results have been found in the literature. It starts to be obvious that the users will need to find a tradeoff solution to optimize the best parameters on a case-by-case basis. To do so, research should be carried out to help users find the optimum printing parameters depending on their needs. It should be noted that this paper looked only at the strength of 3D printed parts, and some users may need high dimensional accuracy (not part of this review paper) which may pertain to another set of optimum parameters. For the improved strength of the part product, the key findings are listed hereafter:

- From the previous discussion, it can be seen that process parameters play a significant role in the determination of the mechanical properties of the part made by FDM. The literature review shows that layer thickness is the most important factor among the studied ones and the information about its effect is contradictory and mainly depends on the type of load applied as well as the raw filament material. Some findings are summarized in Table 1. It can be seen that the data on the effect of the layer thickness are divergent and more work is needed to relate the following parameters and mechanical properties.
- Considering the effect of the infill percentage, all works agree that the increase of infill density increases the strength at break. On the other hand, the same works express the stress at the break as the ratio of load and cross-sectional area without taking into account unfilled space. This is why proper metrics should be developed to account for this issue either by reporting strength per mass (as in the case of Akhoundi, et al. (2019)) or multiplying the stressed area by the infill ratio.
- It also appears that the ratio of the nozzle diameter to the layer thickness may be a major factor affecting the UTS and flexural strength of the FDM printed parts. However, there are currently limited data available on the effect of the nozzle diameter or nozzle diameter/layer thinness ratio, on the impact and compressive strength. Therefore, there is room for further investigation in this direction.
- Extrusion temperature is also able to increase the stress as the bonding between layers and neighbor filaments is facilitated. On the other hand, there is a limit up until which the extrusion temperature can be increased without worsening mechanical properties (Ning, et al. (2016), Guessasma, et al. (2019)).
- Most research indicates lower levels (0° or 15°) of build orientation to be optimal in terms of the tensile strength of FDM parts, whereas the flexural and impact strength properties show different optimal orientations in different studies.
- It was reported that the minimum level (0°) of raster angle improves the tensile strength of FDM parts, while the impact strength can be improved using a 45°/−45° (staggered raster) raster angle.
- The FDM enhancement technique was analyzed based on several existing experiments. The pre-deposition heating method of the extruded layer surface showed a positive impact on the mechanical properties of the printed design. It was also found that the anisotropy effect was reduced after preheating.
- In order to enhance the interlayer bonding temperature and interlaminar strength, the IR lamp heating method was reported to be successful. The printing speed of 3.8 and 5.1 cm/s with two IR lamps of 500 W placed over 8 cm above the deposited layer offers double the fracture energy compared to the nonheated part. A printing speed of 3.8 cm/s and with two IR lamps of 500 W placed over 2.5 cm above the deposited layer also demonstrates a similar result, however, a 1 KW IR lamp placed 1 t 1 cm away from the printed surface results in poor fracture energy due to high intensity of IR that results in degradation of the materials. The idea of IR preheating was to raise the substrate temperature moderately above the glass transition temperature just before the next layer deposition. Lamp configuration and active controlling of substrate temperature need to be investigated further for more insight.
- Incorporation of vacuum in the printing chamber has shown to improve the quality of the printing due to the decrease in heat transfer from the nozzle, hence providing a smooth and slow decrease of the temperature of the polymer that helps to improve the bonding between the layers. Therefore, the tensile strength was found to be increased by 12.83% (from 12.85 MPa to 14.50 MPa) compared to the normal printing conditions.
- One of the current trends in Additive Manufacturing (AM) is to include reinforcement to the polymers in order to enhance the mechanical properties of the printed workpieces such as tensile strength and Young’s modulus. The geometry (such as length) of the reinforcement, their orientation in the matrix and the printing conditions are also significant factors in deciding the strength properties of the FDM parts. According to the literature, the fact that most of the fibers tend to orient along the direction of the extrusion means this inherent preferential orientation resulted in an increased property since this direction of extrusion coincided with the load-bearing direction of the specimens.

Based on the current literature available, the following research gaps are identified:

- As per the literature, most of the studies reported on the tensile properties of the fabricated part, and few studies also reported on flexural strength of the part. In-depth research on flexural strength properties is required to be investigated further in terms of layer thickness, printing speed, extrusion temperature and number of contours. Other than tensile and flexural properties, other properties such as shear stress, impact test to measure the service life of parts also need to be investigated.
- Additionally, research related to linear as well as the circular feature is mostly undertaken, therefore, more complicated shapes with overhangs, gradients and curvature that replicate more real-time problems need to be investigated for strength properties.
- Strength property investigation for dissimilar materials such as plastic and metal are also needed to be done. Due to the different melting points of dissimilar materials, their bonding mechanism also needs to be investigated especially. Additionally, the effect of cavity generated in FDM parts on strength properties needs to be investigated.
- Although infill pattern and infill density have been investigated in the literature, infill pattern with low infill percentage needs to be investigated. In addition, along with other important factors, cooling rate and environmental conditions need to be considered as well.
- Currently, pre-enhancement techniques such as laser heating are mostly in use as per the literature. Other pre-enhancement techniques and post enhancement techniques such as coating also need special attention.
- The FDM part used for medical purposes undergoes a sterilization process, and therefore, the effect of the sterilization technique on the strength properties also needs to be investigated.

## Figures and Tables

**Table 1:** Applications of fused deposition modeling (FDM).

Applications	Reference
Investment casting models	[40]
3D printed models for maxillofacial surgery	[41]
Craniofacial reconstruction and orthopedic inserts.	[42]
Decomposable porous scaffold structures	[38]
Dental repairs, scaffold for organ printing and tissue engineering	[43]
Polycaprolactone/hydroxyapatite artificial bones to imitate goat femur	[44]
Maxillofacial surgery using FDM and poly-jet printing along with finite element analysis (FEA) Simulation and modelling.	[45]
Printing capsules in the pharmaceutical business.	[46]
Scaffold structures for tissue engineering	[47]
Functioning economical prosthetic hand	[48]
Surgical guides for dental application	[49]
Patient-specific bone and respective grafts	[50]
Device for cleft lip and palate (dental field), acoustic prosthesis	[51]
Ornamental industrial objects	[52]
Industrial grade bevel gear	[53]
Textile application	[54]
Sheet metal forming dies	[55]
Personalized lamps	[56]
Electrically conductive plastic patterns	[57]
Components with conductive plastic electronic circuits	[58,59]

**Table 2:** Effect of the layer thickness on the mechanical properties.

Source	Material	Type of the Test	Remarks
Jatti et al. (2019) [60]	PLA	Tensile and flexural strengths, Impact resistance	Increasing infill density increases tensile and flexural strengths of the specimens, due to more material resists the force.
Alafaghani et al. (2017) [64]	PLA	Tensile strength, yield strength, Young’s modulus	Increasing LT increases mechanical properties.
Huynh et al. (2019) [86]	PLA	Tensile strength	Decreasing layer height will increase the strength of the part. Layer thickness is the rank 2 parameter
Sharma et al. (2019) [83]	ABS	Compressive and tensile strength	Increasing the LT decreases the tensile strength, while increases compressive Strength.
Samykano et al. (2019) [75]	ABS	Tensile strength	Layer thickness has no effect and was not a statistically significant parameter
Coogan et al. (2016) [25]	ABS	Tensile strength of the bonds	Layer thickness is the most significant parameter affecting bond strength
Randriguez-Panes et al. (2018) [61]	ABS and PLA	Tensile strength	In the case of PLA, lower layer thickness was desired as it produces the highest strength. In the case of ABS, LT was not significant.

## Data Availability

The data presented in this study are available on request from the corresponding authors.
